# Correction: Kinesin-2 transports Orco into the olfactory cilium of *Drosophila melanogaster* at specific developmental stages

**DOI:** 10.1371/journal.pgen.1011305

**Published:** 2024-05-31

**Authors:** Swadhin Chandra Jana, Priya Dutta, Akanksha Jain, Anjusha Singh, Lavanya Adusumilli, Mukul Girotra, Diksha Kumari, Seema Shirolikar, Krishanu Ray

The Funding statement for this article is incomplete. The correct Funding statement is:

The study was funded by the Department of Atomic Energy, Government of India, TIFR DAE grant nos. 12-R&D-TFR-5.10–100 and RIT4003 (Biol, Chem, Phys & Material Sc.) to KR. SCJ acknowledges support from Fundação Portuguesa para a Ciência e Tecnologia (FCT) grant nos. SFRH/BPD/87479/2012 and PTDC/BIA-CEL/32631/2017 and from Tata Institute of Fundamental Research-DAE (Intramural Project Grant). The funders had no role in study design, data collection and analysis, decision to publish, or preparation of the manuscript.

The Acknowledgments section is incomplete. The correct Acknowledgments are:

We thank Prof. Tomer Avidor-Reiss, University of Toledo, Texas, USA, for the *UAS-Oseg-2*:*GFP* and *UAS-Oseg4*:*GFP* fly stock, Dr. Manish Agarwal (TIFR-H) and Dr. Debasis Das (TIFR) for sharing antibody reagents, and B. Karmakar and R. Phadke for technical help with the instrumentation and experiments. We thank K V Boby and Lalit Borde for their assistance with the confocal and TEM imaging. We especially acknowledge Prof. Monica Bettencourt-Dias, IGC, Portugal, for exceptionally generous support. A part of the experiments was carried out at IGC by SCJ. We thank the IGC Advanced Imaging unit, IGC Electron Microscopy unit and NCBS Central Imaging & FACS Facility for helping us with image acquisition, and the fly facility for assisting us with fly husbandry.
