# Supplementary figures and images for: Kinesin-2 transports Orco into the olfactory cilium of Drosophila melanogaster at specific developmental stages
Source: PLoS Genet. 2021 Aug 19;17(8):e1009752. doi: 10.1371/journal.pgen.1009752 (PMC8407544; doi:10.1371/journal.pgen.1009752)

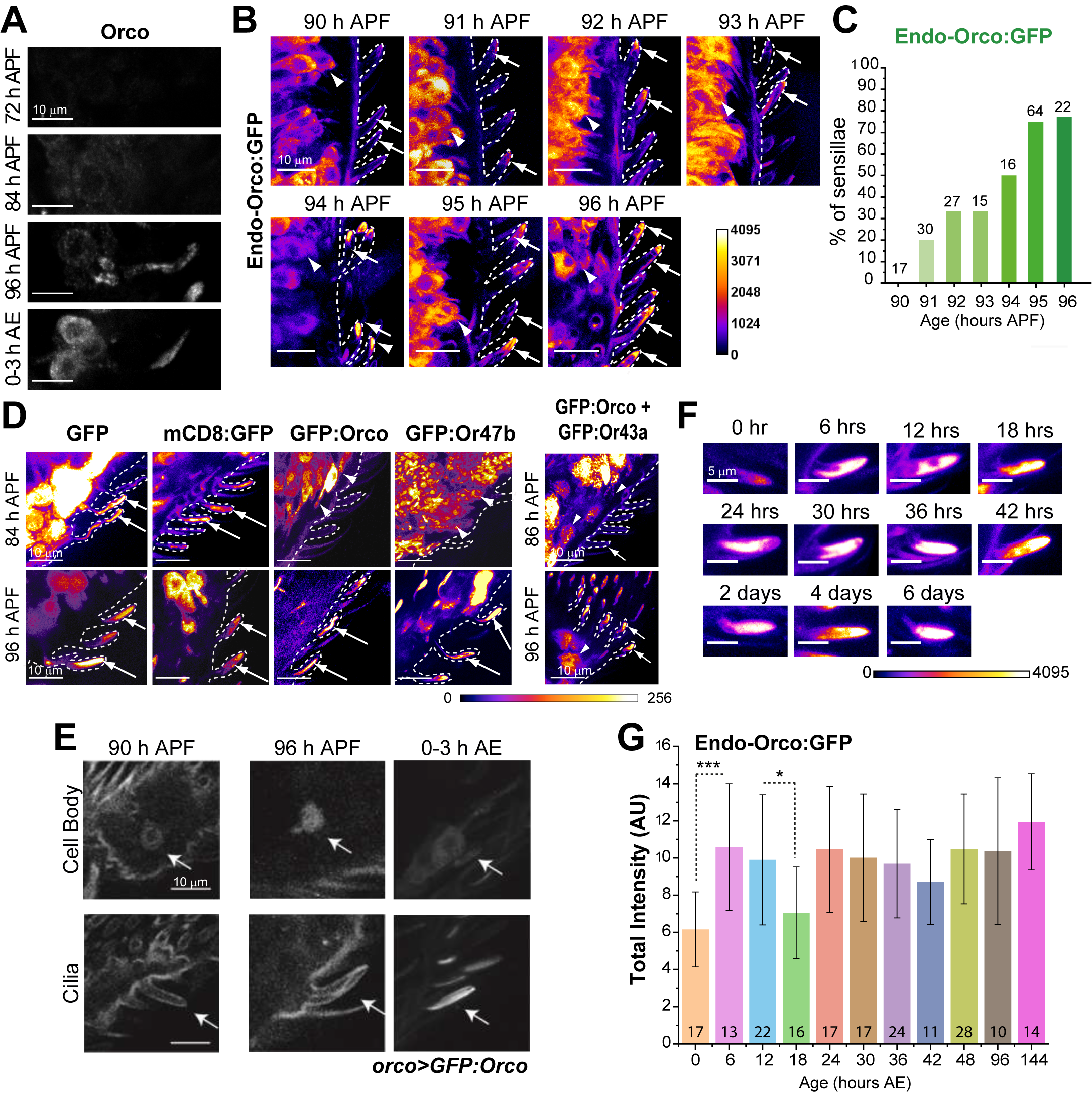

Supplement: S1 Fig — (A) Immunostaining with anti-Orco antibody shows Orco localisation in cilia inside s. basiconica of pupae during 72–96 hours APF and 0–3 hours AE. (B) Endo-Orco:GFP localisation in pupal cilia in s. basiconica from 90–96 hours APF. Arrowheads indicate cell bodies of the OSNs, and arrows mark the ciliary OS. (C) Percentage of sensillae with Endo-Orco:GFP localisation in the ciliary OS of pupal cilia inside s. basiconica during 90–96 hours APF. (D) GFP, mCD8:GFP, GFP:Orco and GFP:Or47b localisations in cilia inside s. basiconica during 84–96 hours APF; and coexpression of GFP:Orco and GFP:Or43a during 86–96 hours APF. All transgenes were expressed using chaGal4. (E) orcoGal4 expression profile in the antennae during pupal (90–96 hours APF) and adult stages (0–3 hours AE). UAS-GFP:Orco was expressed in the OSNs using OrcoGal4 and the cell body and the cilia (indicated by arrows) were imaged at 90 hour APF, 96 hour APF and 0 day AE. GFP:Orco fluorescence becomes visible in the cell body at 90 hour APF and in the cilia from 96 hour APF. (F, G) Endo-Orco:GFP localisation (F) and total fluorescence intensity (mean ± S.D.) of Endo-Orco:GFP (G) in the ciliary OS inside s. basiconica of adult flies during 0–144 h AE. The pairwise significance of difference was estimated using one-way ANOVA test, p-values (*p < 0.05, **p < 0.01, and ***p<0.001) are indicated on the plots. Error bars represent as ± SD. All images are shown in the false colour intensity heat map (FIRE, ImageJ). Scale for images (A), (B) and (C) 10 μm; (E) 5 μm. The number of sensillae (n) quantified are indicated on the graphs. Note: Images shown in panel F and quantification shown in panel G were collected using Olympus FV1200 confocal microscope using different laser power, HV and gain settings as compared to the images of Endo-Orco:GFP shown in other figures, which were collected on FV3000 using uniform laser power and HV and gain settings. Therefore, the intensity values can only be compared amongst the hist [file pgen.1009752.s001.tif]

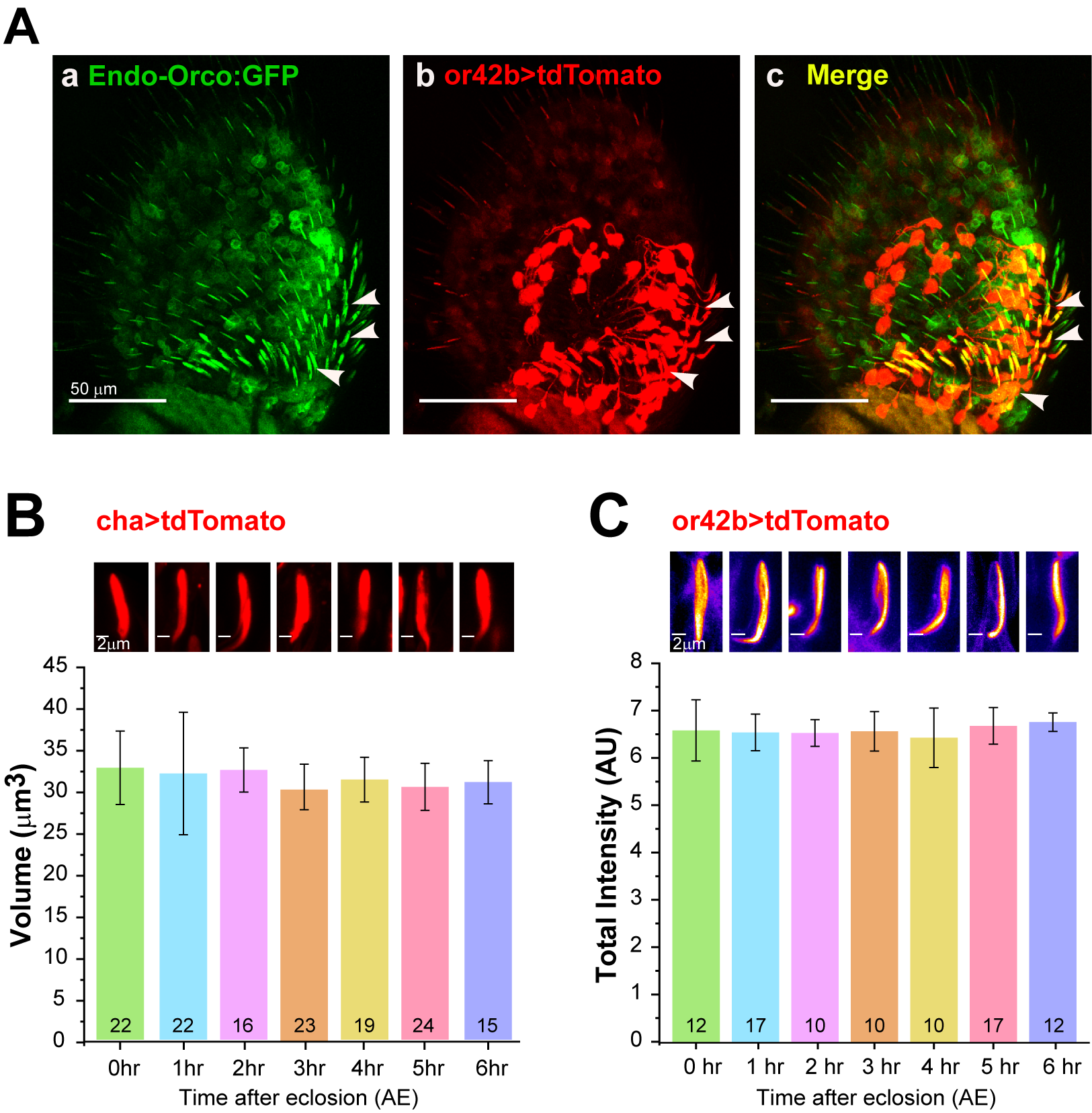

Supplement: S2 Fig — (A) Adult antenna expressing Endo-Orco:GFP and or42b>tdTomato (or42bGal4/UAS-tdTomato) marking the ab1-type sensilla (arrowhead). (B, C) Ciliary volume (mean ± S.D.) marked by tdTomato expressed in all OSNs (B) and the tdTomato fluorescence intensity (mean ± S.D.) in the cilia of Or42b specific OSNs (C) during 0–6 hours AE. The pairwise significance of difference was estimated using one-way ANOVA test, p-values (*p < 0.05, **p < 0.01, and ***p<0.001) are shown on the plots. Images are shown in a false colour intensity heat map (FIRE, ImageJ). Scale bars indicate 50 μm (A) and 2 μm (B, C), respectively. The number of sensillae (n) quantified are indicated on the graphs. (TIF) [file pgen.1009752.s002.tif]

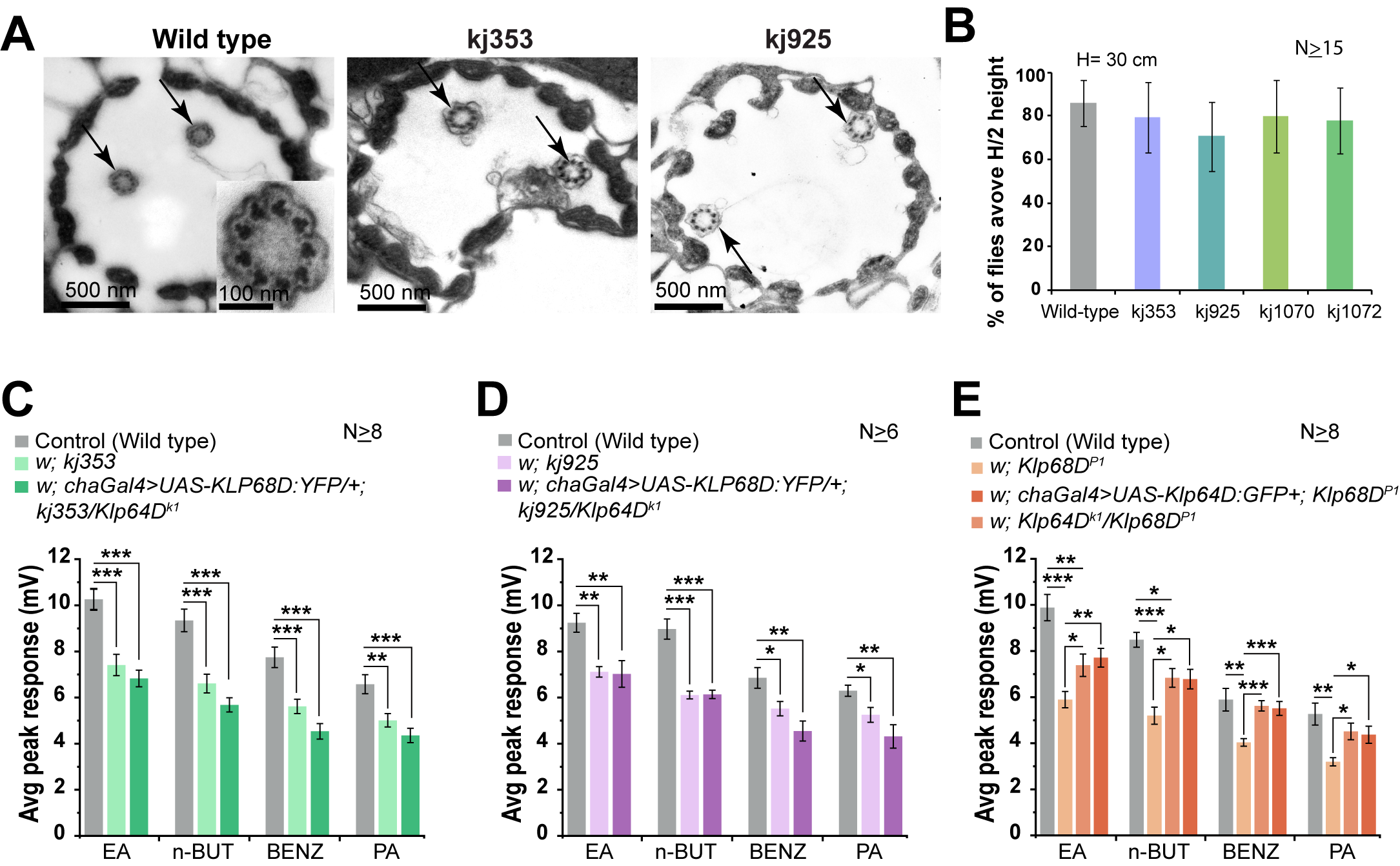

Supplement: S3 Fig — (A-B) The chordotonal cilia are unaffected in homozygous Klp64Dkj backgrounds. TEM images of cross-sections of scolopidia from Johnston’s Organs in wild-type and homozygous mutant antennae (A). Each scolopidium contained two sensory cilia (arrows), with 9+0 organisation of microtubule (inset). The chordotonal cilia, appeared normal in the mutants, play essential roles in proprioception and negative geotaxis. (B) Homozygous Klp64Dkj alleles are coordinated. Negative geotaxis (test of coordination) of wild-type and homozygous kj adults was measured by estimating the relative number of flies above the half-length of a 30 cm cylinder 5 minutes after banging. Homozygous w1118 was used as the wild-type control. It showed that they were coordinated like the wild-type flies. (C-E) EAG response defects of the homozygous Klp64Dkj mutants are not rescued by the OSN-specific expression of the KLP68D transgene. Histograms indicate mean (± S.E.M) electroantennogram (EAG) responses from the antennae of various mutant combinations. Klp64Dk1 is a lethal total-loss-function allele of Klp64D and Klp68DP1 is a hypomorphic, viable, P-element insertion allele of Klp68D. chaGal4 induced expression of UAS-KLP68D:YFP, as well as UAS-KLP64D:GFP in OSNs rescued the cilia development and EAG defects of the homozygous Klp68DP1 (Jana et al., 2011). The data shown in panel E indicate that loss of KLP68D could partly compensate by the overexpression of KLP64D. The number of antennae/flies (N) quantified are indicated on the graphs. (TIF) [file pgen.1009752.s003.tif]

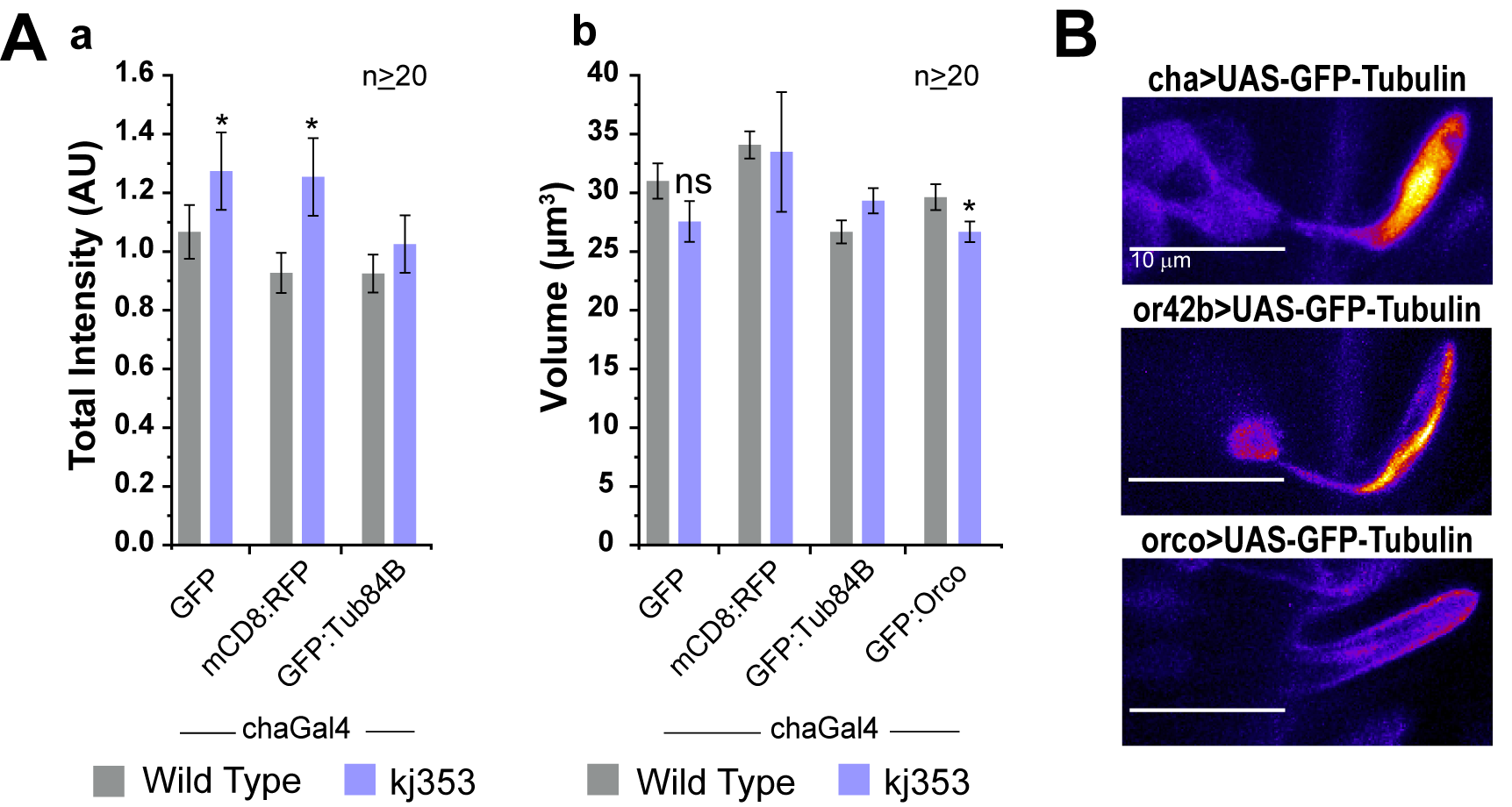

Supplement: S4 Fig — (A) Total fluorescence intensity (a) and volume (mean ± S.E.M.) of ciliary OS (b) inside s. basiconica marked by the soluble eGFP, membrane-associated mCD8:GFP, the cytoskeleton marker GFP:Tubulin84B and GFP:Orco (all driven by chaGal4) at two-day AE in control and homozygous Klp64Dkj353 backgrounds. The pairwise significance of difference was estimated using a two-tailed Student’s T-test, and the p-values (*p < 0.05, **p < 0.01, and ***p<0.001) are indicated on the bars. (B) GFP:Tubulin84B localisation in the ciliary OS of ab1-type s. bascionica in the chaGal4>UAS-GFP:Tubulin84B, or42bGal4>UAS-GFP:Tubulin84B, and orcoGal4>UAS-GFP:Tubulin84B. Note that very little GFP:Tubulin84B is transported into the cilia when expressed in the adult stage by orcoGal4. The expression of chaGal4 starts at 30 hours APF much before the cilia growth, and that of or42bGal4 is found to begin at 60 hours APF. The adult fly emerges at 105 hours APF at 25°C. All images are shown in the false colour intensity heat map (FIRE, ImageJ). Scale for images—10 μm. The number of sensillae (n) quantified are indicated on the graphs. (TIF) [file pgen.1009752.s004.tif]

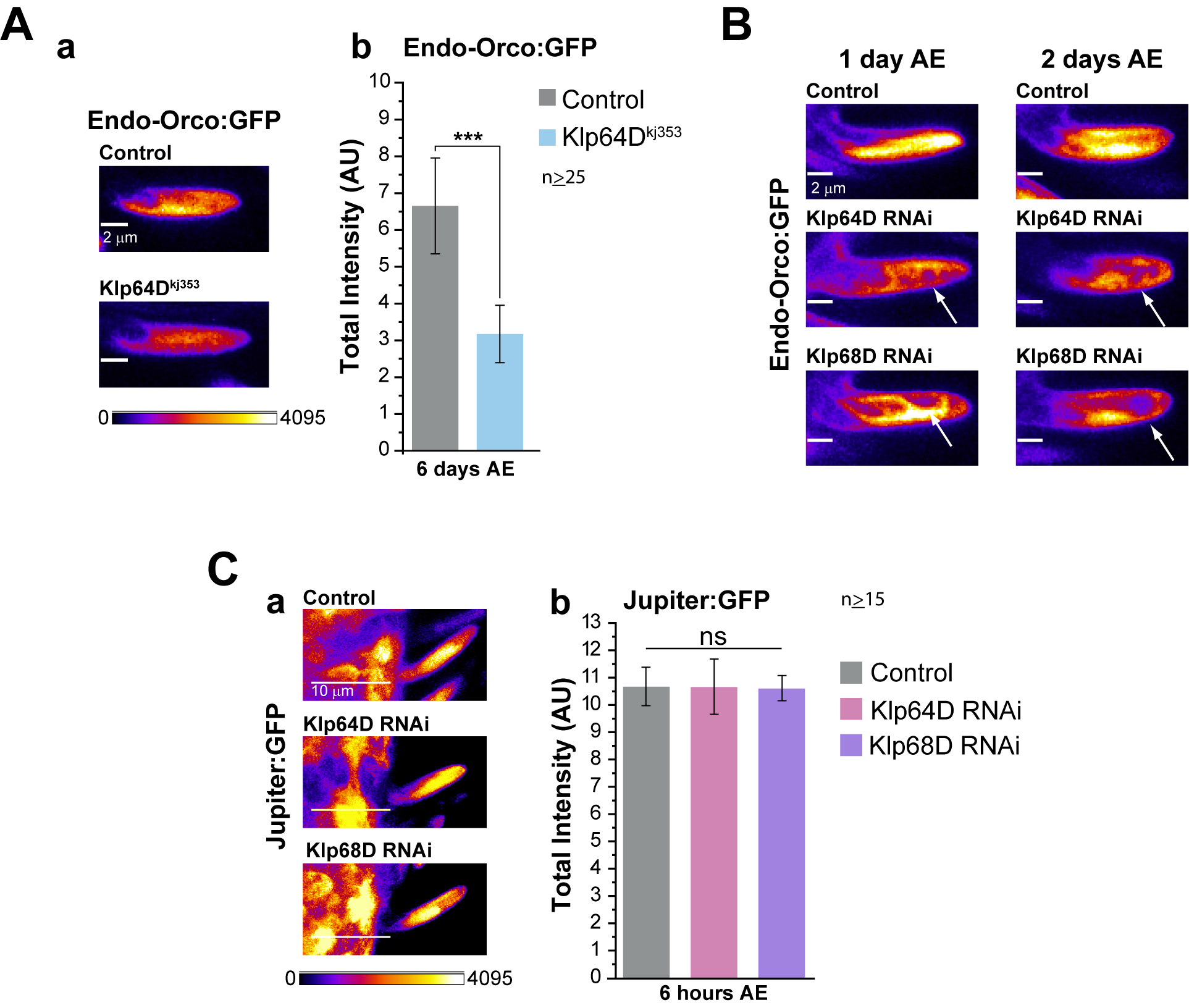

Supplement: S5 Fig — (A) Endo-Orco:GFP localisation inside the ab1-type s. basiconica from Control (Endo-Orco:GFP; +), and homozygous Klp64Dkj353 mutant (Endo-Orco:GFP; Klp64Dkj353) backgrounds at 6 days AE (a). Histograms depict total fluorescence intensity (mean ± S.D.) of Endo-Orco:GFP in Control, and Klp64Dkj353 mutant backgrounds at 6 days AE (b). The pairwise significance of difference was estimated using the two-tailed Student’s T-test, and p-values (*p < 0.05, **p < 0.01, and ***p<0.001) are indicated on the plots. Error bars represent ± S.D. (B) Ciliary morphology is affected due to the prolonged absence of the kinesin-2 subunits in the RNAi backgrounds. Fluorescence micrographs depicting the distribution of Endo-Orco:GFP in the ciliary OS of the Control, Klp64D RNAi and Klp68D RNAi at 1 day and 2 days AE. Since the ciliary structure seemed disrupted (presence of hollow pocket-arrows) at 1 day and 2 days AE, we did not estimate Endo-Orco:GFP levels in the ciliary OS beyond 12 hours AE. (C) Kinesin-2 knockdown does not affect the cytoskeleton of the cilia inside s. basiconica. Jupiter:GFP localisation in the ab1-type s.basiconica from control (orcobGal4/+; Jupiter:GFP/UAS-Dicer), Klp64D RNAi (orcoGal4/UAS-Klp64D RNAi; Jupiter:GFP/UAS-Dicer) and Klp68D RNAi (orcoGal4/UAS-Klp68D RNAi; Jupiter:GFP/UAS-Dicer) background at 6 hours AE (a). Histograms depict total Jupiter:GFP fluorescence intensity (mean ± S.D.) in Klp64D and Klp68D RNAi backgrounds at 6 hours AE (b). The pairwise significance of difference was estimated using the one-way ANOVA test, p-values (*p < 0.05, **p < 0.01, and ***p<0.001) are indicated on the plots. All images are shown in the false colour intensity heat map (FIRE, ImageJ). Scale for images—10 μm (A-a, and C-a); and 2 μm (B). The number of sensillae (n) quantified are indicated on the graphs. (TIF) [file pgen.1009752.s005.tif]

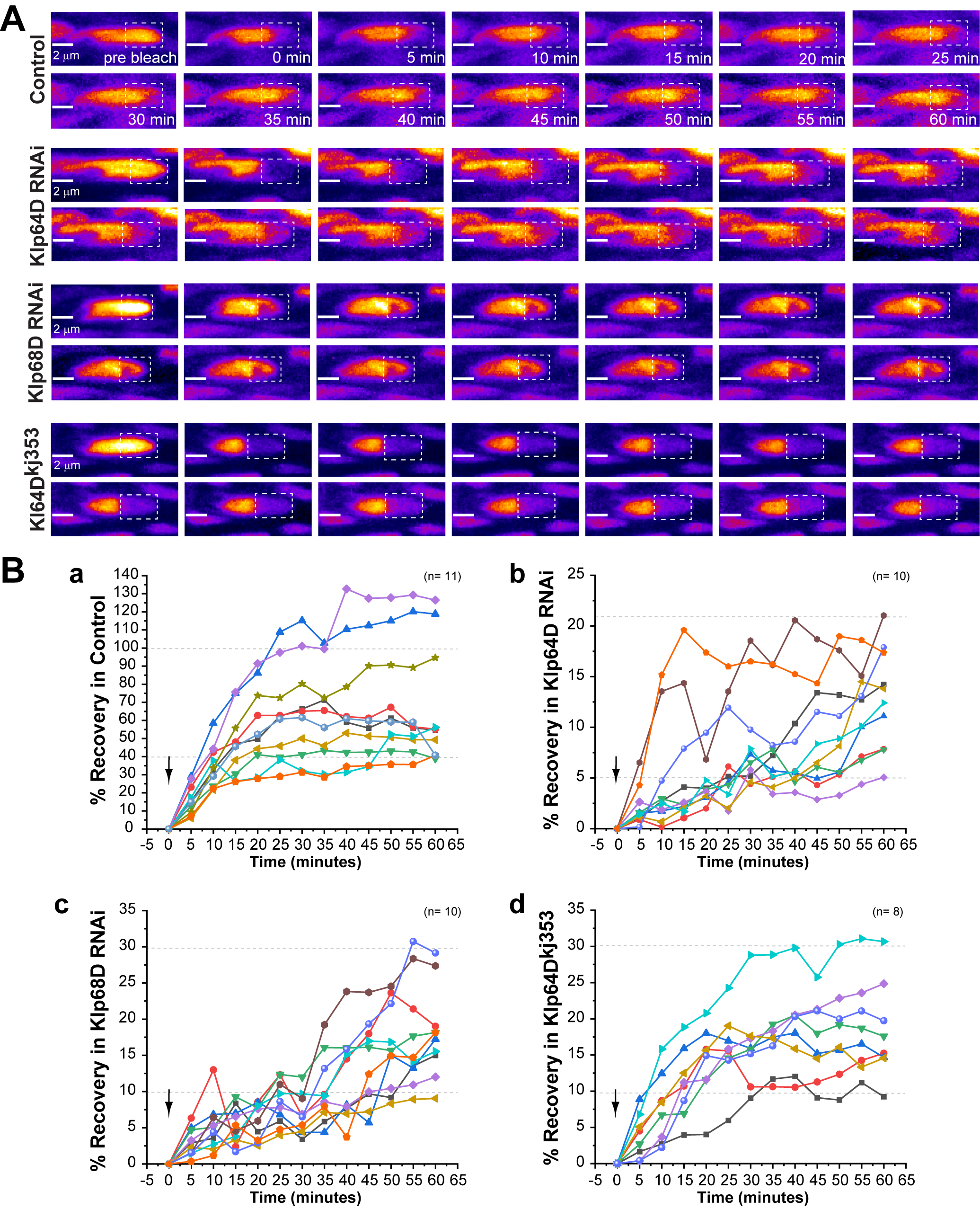

Supplement: S6 Fig — (A) Fluorescence recovery of Endo-Orco:GFP after photobleaching (FRAP) in the ciliary OS in s. basiconica of the control (orcoGal4, Endo-Orco:GFP/+; UAS-Dicer/+), Klp64D RNAi (orcoGal4, Endo-Orco:GFP/UAS-Klp64D RNAi; UAS-Dicer/+), Klp68D RNAi (orcoGal4, Endo-Orco:GFP/UAS-Klp68D RNAi; UAS-Dicer/+) and homozygous Klp64Dkj353 mutant (Endo-Orco:GFP; Klp64Dkj353) backgrounds during 4–5 hours AE. All images are shown in the false colour intensity heat map (FIRE, ImageJ), and scale indicates 2 μm. (B) Relative FRAP profiles are plotted for individual s. basiconica in different genetic backgrounds. Note that the recovery profiles in control were unimodal. It reached saturation after 15 minutes in a majority of cases. However, in a significant number (n = 3 of 11) of cases, a second spurt occurred in the midcourse, which enhanced the recovery beyond 100%, suggesting that Orco is perhaps transported in 15–20 minute spurts during 4–5 hours AE. The loss of kinesin-2 significantly slowed the recovery. The number of sensillae (n) quantified are indicated on the graphs. (TIF) [file pgen.1009752.s006.tif]

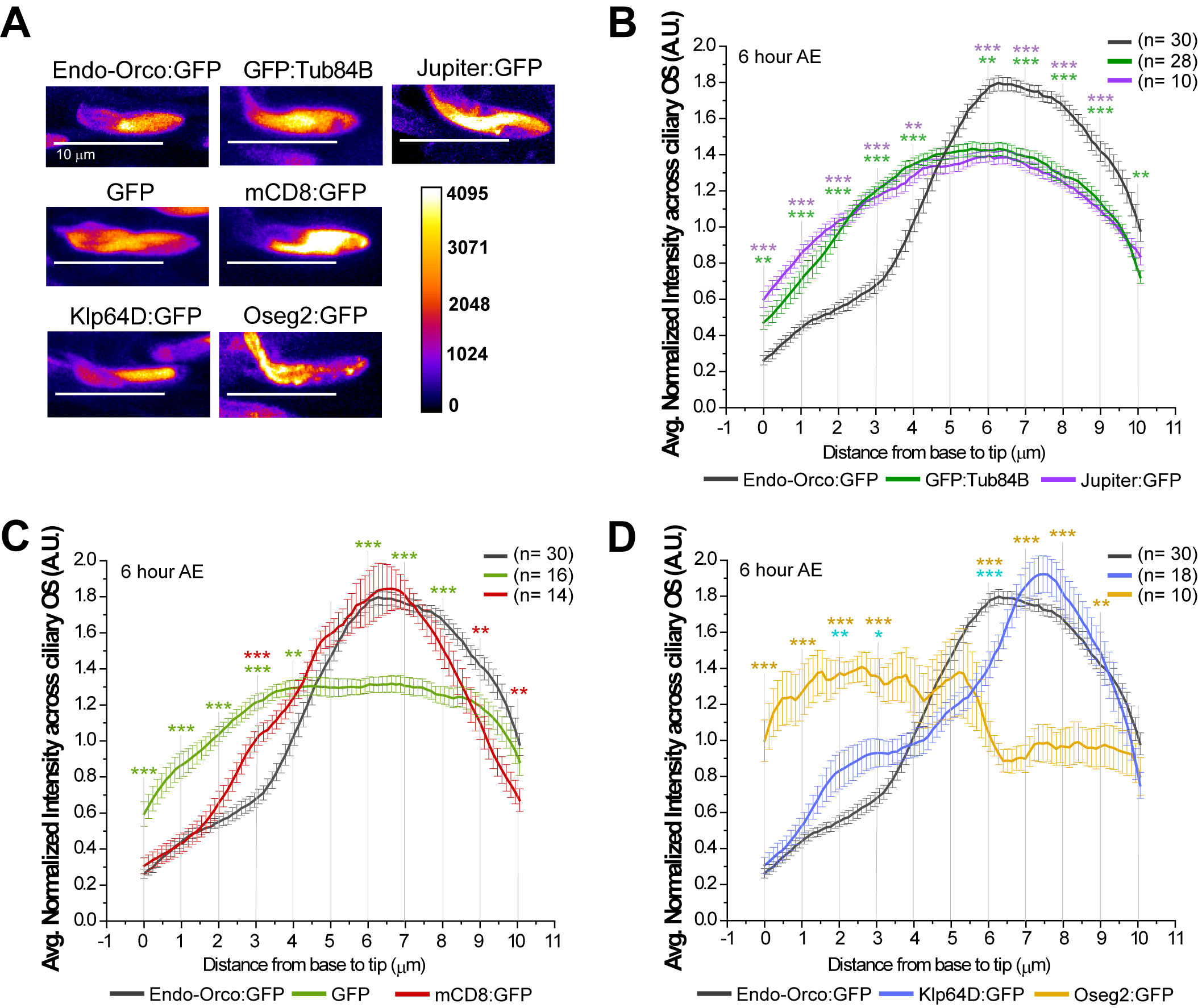

Supplement: S7 Fig — (A) Ciliary localisation of Endo-Orco:GFP, cytoskeleton markers- GFP:Tubulin84B and Jupiter:GFP; soluble protein- eGFP; membrane marker- mCD8:GFP; and ciliary trafficking machinery- Klp64D:GFP and Oseg2:GFP, at 6 hours AE. Except for Jupiter:GFP (protein trap) and Endo-Orco:GFP (fosmid line), all the oher transgene expressions were driven by the chaGal4. (B-D) Plot profiles of the distribution (mean ± SEM) of Endo-Orco:GFP and the other ciliary markers along the ciliary OS inside ab1-type s. basiconica at 6 hours AE. The pairwise significance of difference was estimated at every 1 μm interval along the length of the cilia using one-way ANOVA test, p-values (*p < 0.05, **p < 0.01, and ***p<0.001) are indicated on the plots. All images are shown in the false colour intensity heat map (FIRE, ImageJ). Scale for images- 10 μm. Number of sensillae (n) quantified are indicated on the graphs. (TIF) [file pgen.1009752.s007.tif]

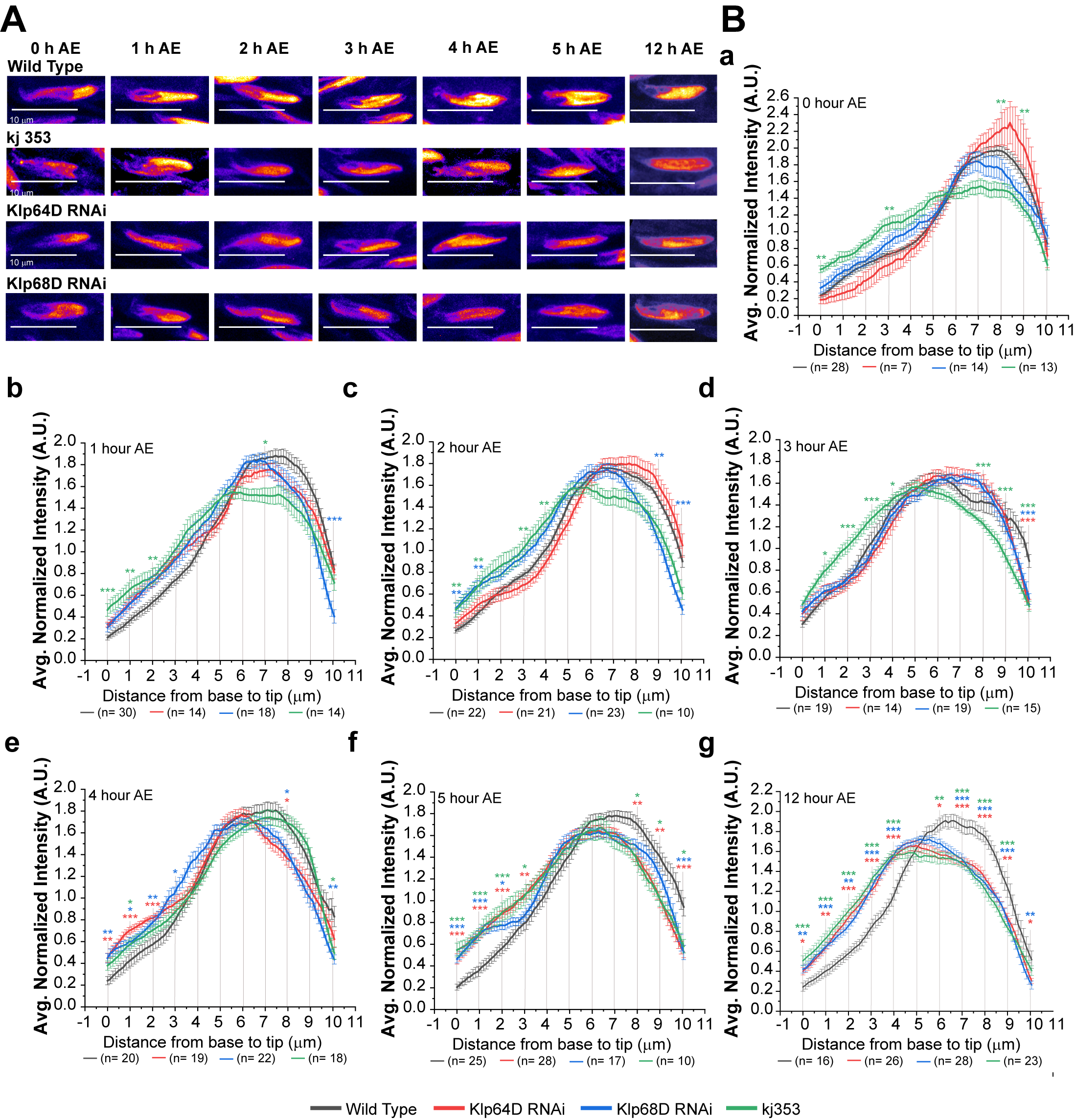

Supplement: S8 Fig — (A) Fluorescence intensity micrographs of Endo-Orco:GFP along the ciliary OS of ab1-type s. basiconica during 0–5 hours AE due to the knockdown of kinesin-2 motor subunits–KLP64D and KLP68D. and in the homozygous Klp64Dkj353 backgrounds, respectively. All images are shown in the false colour intensity heat map (FIRE, ImageJ). Scale for images- 10 μm. (B) Comparison of the relative distribution of Endo-Orco:GFP (mean ± S.E.M) during 0–5 hours AE and 12 hours AE in control, KLP64D RNAi, KLP68D RNAi, and homozygous Klp64D kj353 mutant backgrounds. The pairwise significance of the differences was estimated at a few sample points along the ciliary OS using a one-way ANOVA test; p-values (*p < 0.05, **p < 0.01, and ***p<0.001) are indicated on the plots. The number of sensillae (n) quantified are indicated on the graphs. (TIF) [file pgen.1009752.s008.tif]

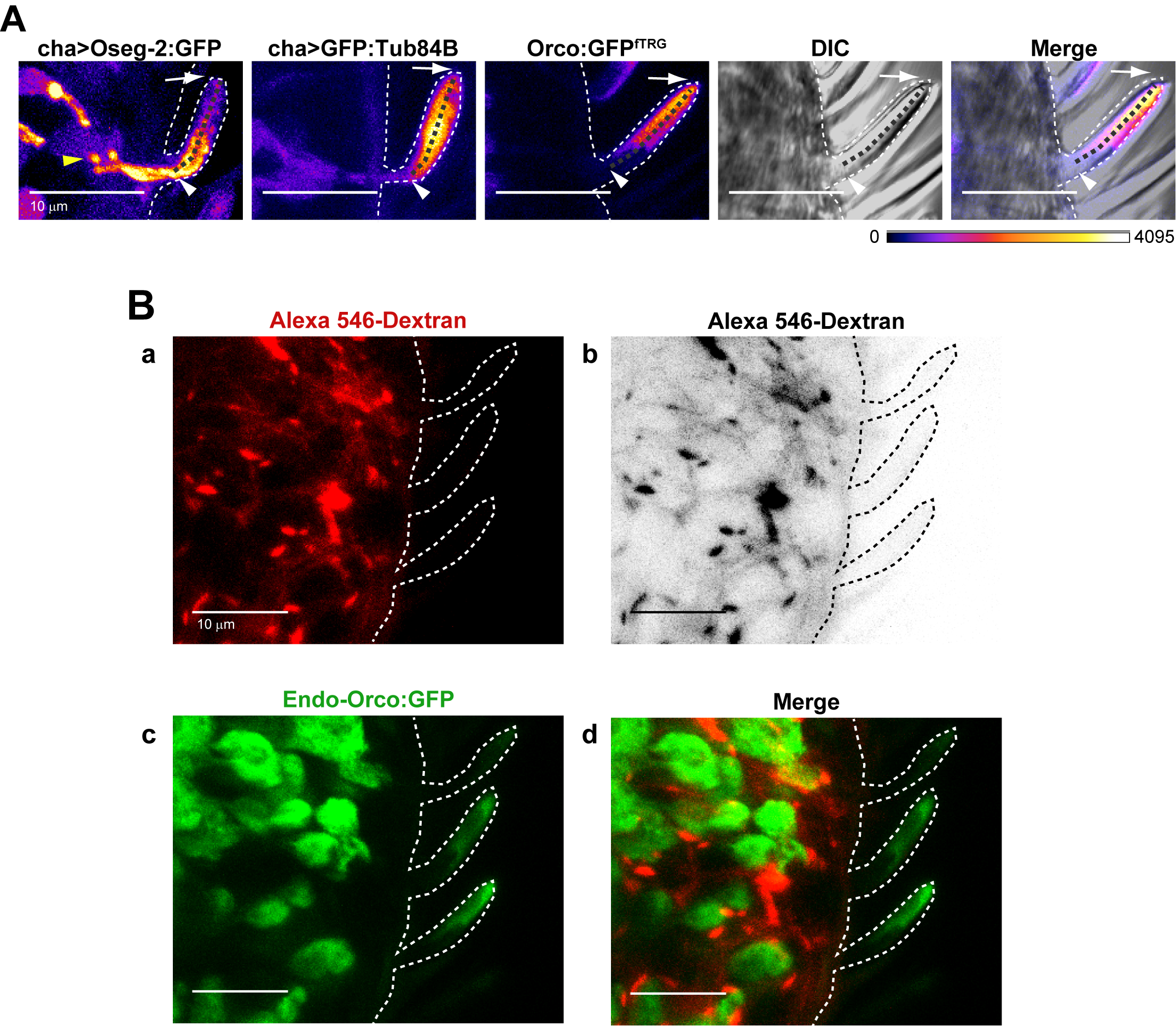

Supplement: S9 Fig — (A) Fluorescence micrographs of cilia in s. bascionica showing localisation of Oseg-2:GFP, GFP:Tubulin84B, Orco:GFPfTRG (Endo-Orco:GFP), DIC of the cuticle of s. basiconica, and merge of DIC and Endo-Orco:GFP. The starting point of the ciliary OS (white arrowheads) coincides with the base of the cuticle shaft and are always marked by a characteristic bend. EndoOrco:GFP mostly localises after this bend (white arrowhead), whereas Oseg-2:GFP was found to localise from the basal body (yellow arrowhead) through the inner segment and up to the middle region of the ciliary OS. The distal-most end of the ciliary OS marks the endpoint (white arrows) for measurement of the plot profiles. The length along which the line segment was drawn to measure the plot profiles is represented by a dark grey dotted line. All images are shown in the false colour intensity heat map (FIRE, ImageJ). (B) Localisation of Alexa 546-Dextran is excluded from the OSNs and sensillae. (a) Alexa 546-Dextran marked the coelom of the third antennal segment. (b) Inverted image of Alexa 546-Dextran indicates its exclusion form the sensillum shaft region. (c) Endo-Orco:GFP marking the OSNs and the s. basiconica. (d) Merge of Alexa 546-Dextran and Endo-Orco:GFP depicting that Dextran is excluded from the lymph surrounding the olfactory sensory cilia innervating the sensillae. (TIF) [file pgen.1009752.s009.tif]
