# Supplementary material for: Kinesin-2 transports Orco into the olfactory cilium of Drosophila melanogaster at specific developmental stages
Source: PLoS Genet. 2021 Aug 19;17(8):e1009752. doi: 10.1371/journal.pgen.1009752 (PMC8407544; doi:10.1371/journal.pgen.1009752)
Supplement: S1 Table — Olfactory responses for third instar larvae from wildtype, Klp64D kj mutants, and Or83b/Orco null (positive control) were measured by performing the larval plate assay. Third instar larvae were placed in the center of petriplate with odour on one half of the plate and diluent on the other half. They were given a choice to move towards either direction. The larvae on each side was calculated and the response index was calculated for each genotype. Klp64D kj mutant larvae did not show any olfactory response defects towards the odours when compared to wildtype. While Or83b/Orco null mutants showed a marked decrease in response, as expected. Note: n-vale indicates total number of runs counted. Each run lasted for 15 minutes and consisted of approximately 20 larvae. (DOCX) [file pgen.1009752.s010.docx]

| Stimulus | Ethyl Acetate (10^-5^) | | | Butanol (10^-2^) | | | Fructose (1 M) | | |
| --- | --- | --- | --- | --- | --- | --- | --- | --- | --- |
| **Genotype** | **Mean** | **S.D.** | **n** | **Mean** | **S.D.** | **n** | **Mean** | **S.D.** | **n** |
| **Canton S** | 0.79 | 0.12 | **118** | 0.61 | 0.14 | **123** | 0.671 | 0.129 | **79** |
| **Kj353** | 0.83 | 0.08 | **32** | 0.69 | 0.13 | **32** | 0.671 | 0.093 | **24** |
| **Kj429** | 0.83 | 0.10 | **27** | 0.65 | 0.12 | **27** | 0.610 | 0.089 | **24** |
| **Kj925** | 0.80 | 0.10 | **35** | 0.63 | 0.13 | **28** | 0.669 | 0.120 | **26** |
| **Kj1072** | 0.83 | 0.11 | **52** | 0.55 | 0.13 | **51** | 0.466 | 0.113 | **37** |
| **Kj1070** | 0.88 | 0.07 | **30** | 0.69 | 0.12 | **32** | 0.678 | 0.096 | **25** |
| **OR83b^1^** | 0.03 | 0.22 | **26** | 0.08 | 0.15 | **21** | 0.760 | 0.090 | **24** |
|  |  |  |  |  |  |  |  |  |  |
