# Supplementary material for: Kinesin-2 transports Orco into the olfactory cilium of Drosophila melanogaster at specific developmental stages
Source: PLoS Genet. 2021 Aug 19;17(8):e1009752. doi: 10.1371/journal.pgen.1009752 (PMC8407544; doi:10.1371/journal.pgen.1009752)
Supplement: S2 Table — EAG values were obtained from 2-days old adult flies of specific genotypes. Values in each cell indicate mean (± S.D.) responses in mV (N≥6) for each genotype. Pairwise significance of differences was calculated with respect to the wild type control values for the respective odours using two tailed Student’s T-test. Significant (*, p<0.1) and very significant (**, p<0.01) differences are indicated in the cells. Color key: red—Ethyl Acetate (EA), blue n-Butanol (n-But) (DOCX) [file pgen.1009752.s011.docx]

|  | | ***Klp64D (recessive, uncoordinated alleles}*** | | | | | | **Control** | |
| --- | --- | --- | --- | --- | --- | --- | --- | --- | --- |
|  |  | ***k5*** | | ***l3*** | | ***l4*** | | ***+*** | |
|  |  | **EA** | **nBut** | **EA** | **nBut** | **EA** | **nBut** | **EA** | **nBut** |
| ***Klp64D (jump response defect, viable alleles )*** | ***kj353*** | **6.7±0.5**** | **4.2±0.2**** | **6.2±0.3**** | **3.9±0.5**** | **7.5±0.8**** | **5.4±0.6*** | **9.0±0.6** | **7.8±0.9** |
|  | ***kj429*** | **6.1±0.4**** | **4.3±0.4**** | **6.2±0.3**** | **4.2±0.5**** | **5.0±1.5*** | **4.1±1.2*** | **8.3±0.6** | **6.4±0.4** |
|  | ***kj925*** | **4.5±1.2**** | **3.0±0.6**** | **6.0±0.3**** | **3.6±0.4**** | **6.2±0.8**** | **3.8±0.7**** | **9.5±1.3** | **7.2±1.2** |
|  | ***kj1070*** | **5.7±0.6**** | **3.0±0.8**** | **5.6±0.4**** | **3.7±0.4**** | **6.3±1.4**** | **3.5±0.5**** | **8.5±0.9** | **6.5±0.6** |
|  | ***kj1072*** | **5.9±0.8**** | **3.6±0.8**** | **5.5±0.3**** | **3.8±0.3**** | **6.3±0.7**** | **3.5±0.3**** | **9.7±0.6** | **7.6±0.9** |
|  | **Rescue Genotypes** | | | | | | | **EA** | **n-But** |
| ***Transgenic Rescue*** | ***w; Gal4^OR83b^/UAS-KLP64D; kj353*** | | | | | | | **8.7+0.9** | **7.9+0.3** |
|  | ***w; Gal4^OR83b^/UAS-KLP64D; kj925*** | | | | | | | **10.2+1.3** | **9.3+1.3** |
|  | ***w; Gal4^SG18.1^/UAS-KLP64D; kj353*** | | | | | | | **9.5+0.7** | **8.1+1.3** |
|  | ***w; Gal4^SG18.1^/UAS-KLP64D; kj925*** | | | | | | | **10.4+0.3** | **9.1+0.8** |
