# Supplementary material for: Kinesin-2 transports Orco into the olfactory cilium of Drosophila melanogaster at specific developmental stages
Source: PLoS Genet. 2021 Aug 19;17(8):e1009752. doi: 10.1371/journal.pgen.1009752 (PMC8407544; doi:10.1371/journal.pgen.1009752)
Supplement: S3 Table — The fly stocks, genotypes and source information is listed in this table. (DOCX) [file pgen.1009752.s012.docx]

| **Fly stocks Used** | **Nature** | **Reference** |
| --- | --- | --- |
| *chaGal4* | Transgene, recombinant Gal4 under *Cha* promoter, expresses in cholinergic neurons | Salvaterra and Kitamoto, 2001 |
| *orcoGal4* | Transgene, recombinant Gal4 under *Or83b* promoter, expresses in odour sensing neurons | Wang et al., 2003 |
| *UAS-GFP* | Recombinant GFP transgenes expressed under Gal4/UAS promoter | Spana, 1999.9.27, P[34] constructs and insertions from Eric Spana. |
| *UAS-GFP:Tubulin84B* | Transgene, N-terminal fusion of eGFP to tubulin84B, cloned under the UAS enhancer elements. | Avidor-Reiss et al., 2004 |
| *UAS-GFP:Orco* | Transgene, N terminal fusion of eGFP to Orco insert with ORF and 3’UTR corresponding to nucleotide 168-1917. | Benton et al.,2006 |
| *UAS-GFP:OR43a* | Transgene, N terminal fusion of eGFP to Or43a ORF and 3’UTR | Benton et al.,2006 |
| *UAS-GFP:Or47b* | Transgene, N terminal fusion of eGFP to Or47b ORF and 3’UTR | Benton et al.,2006 |
| *Orco:GFP^fTRG^* | Fosmid line expressing C terminal fusion of GFP to Orco, under its endogenous promoter. | Sarov et al., 2016 |
| *UAS-mCD8:GFP* | eGFP Fusion between mouse lymphocyte marker CD8 and the green fluorescence protein | Lee and Lou et al., 1999 |
| *UAS-mCD8:RFP* | Recombinant, mouse lymphocyte marker CD8 and the red fluorescence protein | Yang et al., 2008 |

| **Fly stocks Used** | **Nature** | **Reference** |
| --- | --- | --- |
| *Jupiter:*GFP | Protein trap, GFP inserted in place of first intron of the endogenous Jupiter Protein. | Karpova et al., 2006 |
| *UAS-Klp64D* | Recombinant full-length KLP64D Transgene | Ray et al., 1999 |
| *UAS-Klp64D:GFP* | Recombinant full-length KLP64D Transgene with C-terminal GFP fusion | Jana et al, 2011 |
| *UAS-Klp68D:YFP* | Recombinant full-length KLP68D Transgene with C-terminal YFP fusion | Jana et al, 2011 |
| *Klp64D^k1^* | Amorphic allele, uncoordinated adults, missense mutation (G13*) | Ray et al., 1999 |
| *Klp64D^k5^* | Amorphic allele which carrying a missense mutation (Glu551-Lys) at the C-terminal of the Klp64D stalk domain | Ray et al., 1999 |
| *Klp64D^kj353^*  *Klp64D^kj925^*  *Klp64D^kj1072^* | Hypomorphic *Klp64D* allele; fails to complement the odour reception defects with lethal/uncoordinated, *Klp64D* alleles, such as *Klp64D^k1^*, *Klp64D^k5^* and *Klp64D^k4^*. | Anjusha K, Jana SC and Ray K, this manuscript. |
| Klp68D^EY00199^ or Klp68D^P1^ | Hypomorph, Recombinant P-P{EPgy2}<w+>element inserted in the 5’UTR. | Bellen et al., 2004 |
| *UAS-Oseg-2:GFP* | eGFP Fusion between *Drosophila* orthologue of the IFT-72, Oseg-2 | Avidor-Reiss et al., 2004 |
| *UAS-Oseg-4:GFP* | eGFP Fusion between *Drosophila* orthologue of the WDR35, Oseg-4 | Avidor-Reiss et al., 2004 |
