# Supplementary material for: Kinesin-2 transports Orco into the olfactory cilium of Drosophila melanogaster at specific developmental stages
Source: PLoS Genet. 2021 Aug 19;17(8):e1009752. doi: 10.1371/journal.pgen.1009752 (PMC8407544; doi:10.1371/journal.pgen.1009752)

Figure 6: Western blots

Figure 6b

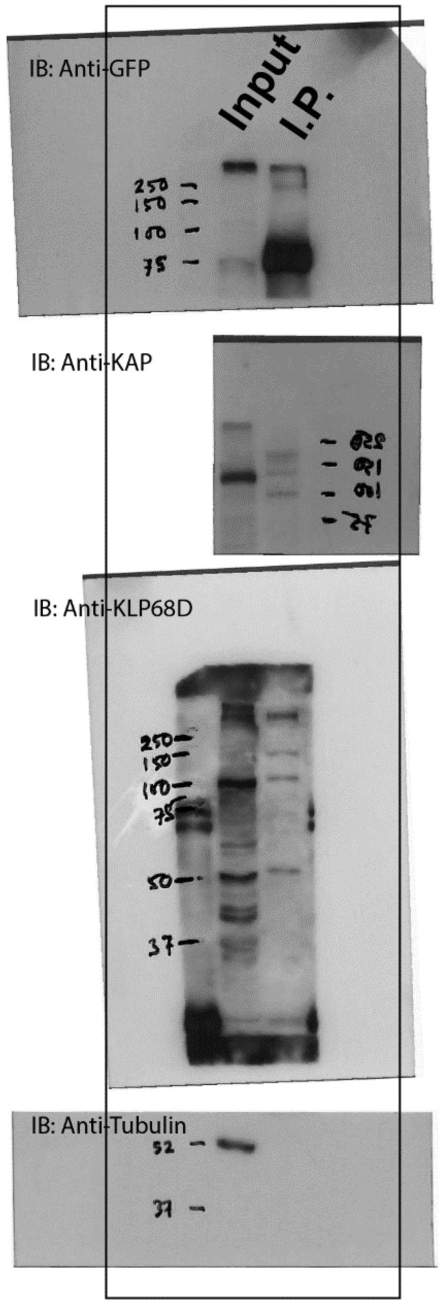

Figure 6c

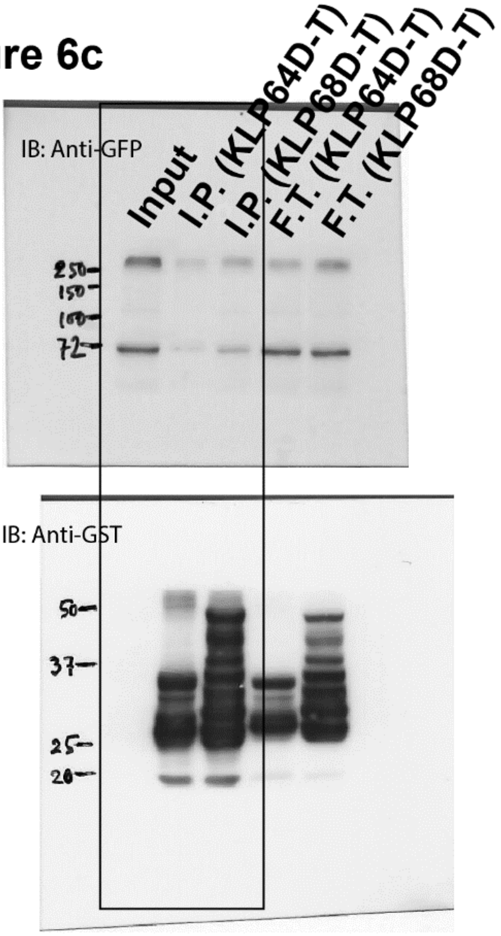

IP - Immun precippitation
IB - immunoblot staining
FT - flow through

Figure 6d: Loading order from the marker lane (right → left) : #1) input, #2) bead control, #3) GST, #4) GST-KLP68D-Tail, #5) GST-KLP64D-Tail

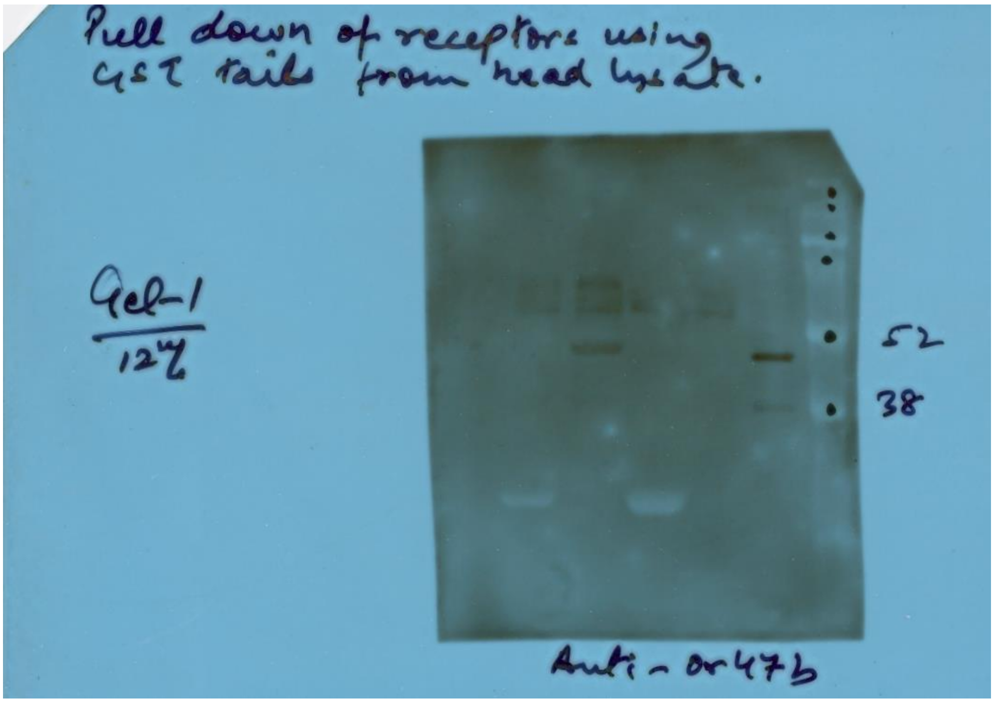

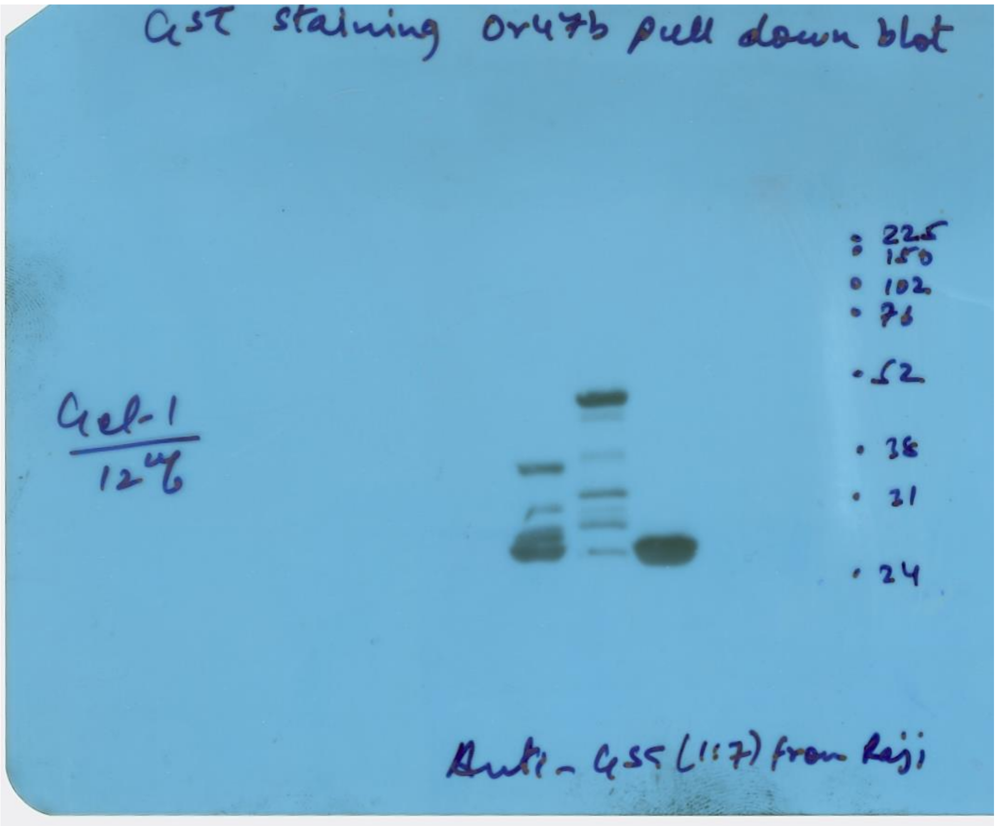

Supplement: S5 Data — (PDF) [file pgen.1009752.s017.pdf]
