# Supplementary material for: Kinesin-2 transports Orco into the olfactory cilium of Drosophila melanogaster at specific developmental stages
Source: PLoS Genet. 2021 Aug 19;17(8):e1009752. doi: 10.1371/journal.pgen.1009752 (PMC8407544; doi:10.1371/journal.pgen.1009752)
Supplement: Attachment — Submitted filename: List of chanegs and resposne to reviewers.docx [file pgen.1009752.s018.docx]

**List of Changes made**

**Introduction:**

1. From line 65 to line 101, the introduction has been extensively revised to provide sufficient context regarding the transport of receptor proteins in the cilia especially with respect to Smoothened and Gli, as the issue raised by the reviewers.

**Results:**

2. From line 233 to line 267, we have revised the explanation of the results mentioned in the Figure 3D-F, as per the issue raised by the reviewer 2.
3. We have included the data of Endo-Orco:GFP localisation in ciliary OS in Control, Kinesin-2 knock down and mutant backgrounds at 12 hours AE (Figure 4E). An extension of this result of Endo-Orco:GFP localisation in the Ciliary OS in Control and Klp64D kj353 mutant at 6 days AE has been included as part of the Supplementary Figure S5A. We have also looked at the localisation of Endo-Orco:GFP in the ciliary OS in the kinesin-2 knockdown background at 1-day and 2-day AE, included in Supplementary Figure S5B.
4. We have also included the results of the new FRAP experiment to estimate if transport of Endo-Orco:GFP is impaired in the kinesin-2 knock down and mutant backgrounds. The recovery of Endo-Orco:GFP at 60 mins after photobleaching is mentioned in figure 4F. The entire recovery profiles are depicted in the Supplementary Figure S6A-B.
5. We have included an entire main figure for depicting that IFT components like Oseg2 and NompB may not be required for Orco transport as part of the Figure 5B-C.
6. We have also included the plot profile of Endo-Orco:GFP distribution across the ciliary OS in kinesin-2 knock down and mutant backgrounds at 12 hours AE as part of Supplementary Figure S9G.
7. We have revised the representative image for the western blot of Orco interaction with tail domains of the kinesin-2 subunits, Figure 6C.
8. We have also included the colocalisation data of Endo-Orco:GFP and Klp68D:YFP and their respective plot profiles as part of Figure 7G-H.

**Discussion:**

9. Line 384 to line 400, the discussion has been revised extensively to incorporate the explanation for the new results included in the current draft of the manuscript.

**Materials and Methods:**

10. Detailed protocol for the Jump Assay, FRAP (Fluorescence Recovery After Photobleaching) assay, and imaging conditions for the estimation of localisation of Endo-Orco:GFP and KLP68D:YFP has been added.

**Figures:**

11. Missing scale bars are added in all the figure panels with appropriate labels
12. N values are added on the plots and histograms.
13. Additional figure panels are added in figures - 4E-F, 5C-D, 7G-H, S5A-B, S6, and S8G.

**Figure Legends**

14. All figure legends are revised to conform to an uniform style.
15. Additional legends are added for new panels in figures - 4E-F, 5C-D, 7G-H, S5A-B, S6, and S8G.

Entire manuscript is carefully edited to correct for all types of typographic and grammatical errors.

Authors’ response to reviewer’s comments:

Reviewer #1: This manuscript was an absolute joy to read. The authors show that the heterotrimeric kinesin 2 is responsible for the post-IFT trafficking of the odorant receptors into the distal olfactory cilium, in two specific phases in early adulthood. The experiments are well designed and systematically performed to the conclusions clear and indisputable.

I have only very small comments and a number of typos and grammatical changes that the authors should consider.

Author response: We are gratified by the appreciation and feel that all the hard work put in by all of us are genuinely justified. We hope that the reviewer will find the revised version appropriate for publication

Page 2, abstract. Line 20. I suggest to add “The”. Thus, “The cilium, the sensing centre….”
Author response: We thank the reviewer for the suggestion. We have revised the statement as the following,” The cilium, the sensing centre for the cell, displays an extensive repertoire of receptors for various cell signalling processes.”

Line 31. Delete “a”. “…the loss of critical components….”
Author response: We thank the reviewer for the suggestion. The article is deleted.

Page 3, Author Summary. Line 39. “organelles” should be plural in agreement with “Cilia”.
Author response: We thank the reviewer for the suggestion. We have corrected the plural-singular mismatch.

Line 42. Delete “the”. “…such as photoreception,…”
Author response: We thank the reviewer for the suggestion. The article is deleted.

Page 4. Line 75. “direction” should be singular.
Author response: We thank the reviewer for the suggestion. The entire section is revised as suggested by Reviewer #2.

Page 5. Line 102. By “unprecedented”, do the authors mean “described here for the first time”? Or do they mean that such temporal shifts do not happen earlier in development.
Author response: Indeed, we meant it that way. However, we now realise that such an emphasis is not necessary in the introduction. Therefore, we have changed the statement with the following phrase - “in two short episodes after the adult flies eclose from pupae.”

Line 106. “associates” should be “associate”, for verb agreement.

Author response: We thank the reviewer for picking up this error. It is now rectified in the revised version as the following,” Also, we used affinity chromatography to demonstrate that Orco and OR47b could associate with the C-terminal tail domains of the kinesin-2 motor subunits” (Line 114-116).

Page 6. Line 112. Insert "types" after "sensilla".

Author response: We thank the reviewer for the suggestion. The statement is now revised as the following, “*Drosophila* antenna contains three morphologically distinct typed of cuticular projections, called *sensilla*, each innervated with the cilia of 2-4 OSNs [22, 25].” (Lines 123-124)

Page 7. Line 133. “its” should be “their” for agreement with the plural “sensilla”.

Author response: We thank the reviewer for the keen reading of our manuscript. We have corrected the verve disagreement.

Line 147. I expect the authors will want to remove the reference to Jain’s thesis.

Author response: We agree and removed the thesis citation because the relevant data is presented in Figure S1.

Page 8. Line 164. I could not find any description in the methods section for how the jump test was performed for the screen. The figure shows an outline, but the experimental details should be included in the methods sections. For example, what odorant was used? How was it presented? How often? How was the scoring done?

Author response: We thank the reviewer for pointing out this mistake. The details are now added to the methods section. We have also rectified the erroneous figure calls according to the new organisation of supplemental data.

Line 169. I believe the data mentioned here are in Figure S3, not S2.

Author response: We thank the reviewer for pointing out this mistake. The figure call is now revised as per the new supplemental figures.

Page 9. Line 180. Spelling of “coeloconica”.
Author response: We have corrected the typo.

Lines 182-184. The statement that the EAGs “indicated that the loss of kinesin-2 could suppress odour receptor localisation” is over-stated. This should be presented as an inference, rather than as a conclusion. For example, the authors may wish to change “indicated” to “suggested”.

Author response: We agree with the comment and revised the statement as the following, “These results suggested that the KLP64D function is necessary to develop odour reception in different types of olfactory cilia.”

Page 11. Line 223. Stating the question in the negative is a bit awkward, and suggests a preconceived conclusion. I suggest “To test whether endogenous Orco transport by kinesin-2 depends on IFT, …”
Line 225. Delete “the”: “…just before eclosion”.

Author response: We have now revised the entire section and divided the results into separate sections describing the role of Kinesin-2 and IFT in the process with the following sub headings – “Orco entry and transport into the ciliary outer segment requires kinesin-2” and “Orco localises in the cilia independent of IFT172/Oseg2 and IFT88/NompB”. A new set of data is added in Figures 4E and 5C to strengthen the conclusions. As suggested by the reviewer, we have removed the statement and replaced it with “To assess the function of kinesin-2 in the bi-phasic increment of Orco during 0 – 6 hours AE..” in line 253 of the revised manuscript.

Line 227. Remove reference to Jain’s thesis? Or include the citation in the bibliography.

Author response: The statement is now revised, and we have removed the citation. The data is presented in Figure S1.

Line 236-237. The data are in Figure S5, not S4.

Author response: We have now rectified the figure call according to the current organisation of the supplemental Figures.

Line 241. Again, S5.

Author response: We have now rectified the figure call according to the current organisation of the supplemental Figures.

Page 12. Line 258. Verb agreement: “GFP:Orco and OR47b were selectively…”

Author response:: We have now rectified the error in the following manner, “..a relatively more prominent level of GFP:Orco (Figure 6C) and OR47b, respectively, were pulled-down..”

Page 13. Lines 282-283. The authors point to the similarity of Endo-Orco:GFP and KLP64D:GFP. However, in the figure, KLP64D:GFP does appear to be even more distal than Endo-Orco:GFP. This point does not seem to be addressed by the authors, even in the discussion. Is it possible that this difference could be explained, for example, by the idea that when Orco is released in the distal cilium membrane, it can drift proximally, while the motor protein does not? This would seem to be an important point to address in the discussion.

Author response: We agree that this observation needs a discussion. To test this idea, we conducted a FRAP study in the wild-type and kinesin-2 mutant backgrounds. The FRAP data added in Figures 4E and S6 suggests that it is unlikely that Orco diffuses in the cilium because the Orco recovery into the distal domain is very slow in the wild-type background, and the kinesin-2 knockdown slowed it even more significantly. Also, the colocalisation data added in Figure 7G-H further illustrated that Orco and the motor enrich in partly overlapping domains. With the help of this new additional evidence, we have added a new postulate in the result section in line 338-341 as the following: “…a fraction of ciliary kinesin-2 is engaged in delivering Orco to its parking zone at the distal OS. The anterograde motors are known to inactivate after reaching the distal ends of the cilia and flagella, which may account for the increased accumulation of the kinesin-2 motor subunits at the distal tips.” The tip accumulation of KLP64D:GFP and KLP68D:YFP helped to clearly distinguish the tip compartment as separate from that of the Orco domain (Line 341-343) .

Page 14. Line 304. “…distribution of in the…” needs to be corrected.
Line 306. The word “microtubules” seems to be missing: “…fewer singlet branched microtubules in the…”

Author response: We thank the referee for pointing out this error. We have now corrected the statement as the following, “The ciliary OS contained fewer branches bearing singlet microtubules in the *Klp64D^k5^* homozygous background.”

Line 307. “responses” in the plural.
Line 312. “observations” in the plural.
Author response: We thank for pointing to this error. We have now corrected both statements.

Page 15. Line 325. “helps” for verb agreement.
Author response: We thank for pointing to this error. We have now corrected both statements.

Page 16. Line 340. Spelling of “Orco/ORx”

Author response: It is the correct form of the expression.

Page 18. Lines 386-389. These last 2 sentences of this section do not belong here, as they are results and conclusions. They should be moved to the appropriate sections.

Author response: This experiment was done to clarify the BODIPY loading method. We considered it as the control for the methodology and placed it with the method. Placing it in the results section appeared to break the flow and distract the argument. Therefore, we request the reviewer to allow it to be kept it in its original place. We have now reorganised the supplemental figures and placed this information as Figure S9 at the end of the order.

Line 388. Spelling of “BODIPY”
Author response: We thank the reviewer for pointing to this error. We have now changed the fonts to all caps.

Page 26. Figure 1 legend. Line 630. “show” for verb agreement.
Author response: We have corrected the verb agreement error.

Page 29. Line 665. “driven by orcoGal4 (i) and chaGal4 (ii)”. Is it possible for the authors to switch these 2 subpanels, for parity with part a), where chaGal4 is presented first, then orcoGal4?

Author response: The subpanels are switched as suggested in the revised figure and legend.

Line 666 and line 669 on the next page. Please include the (i) and (ii) indicators for the subpanels. Otherwise it looks strange to have “c)” twice.

Author response: We agree and shifted the panels, and revised the statement as suggested.

Page 34. Bottom of panel f. Spelling of “BODIPY” needs to be corrected.
Author response: We thank the referee for pointing to this error. We have now changed the fonts to all caps.

Page 42. Line 770. Spelling of “scolopidia”.
Line 771. Spelling of “scolopidium”

Author response: We thank the referee for pointing to this error. We have corrected the spelling.

Line 773. “anti-geotropic walk” seems like unconventional terminology. “negative geotaxis” is more conventional, as used in the next line.
Author response: We agree and made the changes as suggested.

Page 44. Line 787-788. The scale bar reference should be moved to part a) of the legend, as there are no scale bars in panel b).
Author response: We apologise for this slip. We have now added scale bars to all figures.

Page 46. Line 796. Delete "in": "unaffected upon adult specific".

Author response: The statement is replaced.

Reviewer #2: In this manuscript Jana et al., report their efforts to understand the mechanisms that localise the odor-receptor coreceptor (Orco) into the cilia of the olfactory sensory neurons. The authors find that kinesin-2 transports Orco into the cilium, and this activity is independent from its function in IFT. In addition, they show that Orco enters cilia of the olfactory sensory neurons at a specific developmental time following the eclosion, in two separate bursts. Once in the cilium, kinesin-2 restricts Orco’s localisation to a specific ciliary subdomain.
Using genetic approaches, the authors were able to dissect the function of kinesin-2 to study its role in transport of transmembrane receptors into the cilia, independently of IFT and cilia development. Although, this study does not provide much mechanistic insight into how kinesin-2/Orco entry is regulated, the authors show compelling evidence that kinesin-2 plays a complex role in cilia of olfactory neurons. As ciliary signaling and establishment of unique signaling domains within ciliary compartments is still poorly understood, the work by Jana et al., provide valuable insight into the transport and localisation of a transmembrane receptor, Orco into the cilia of olfactory neurons in adult flies.

However, some revisions and additional experiments are needed before the manuscript can be accepted for publication.

1. Introduction needs revisions, the authors should provide sufficient context for presented background, for example while explaining Smo transport, the authors state: ‘Whereas the kinesin-family motor Costal2/Kif7, which is immotile and does not bind to either Smo or Gli, is implicated in establishing the signalling-competent […]’, the role of Gli in Smo’s transport/function is unclear.

Author response: We appreciate this comment and have now revised the middle portion of the introduction to introduce the context and streamline the flow argument. It also highlights the central motivation behind this study.

2. All abbreviations should be explained immediately as they appear.

Author response: We appreciate this comment and made the necessary changes throughout the manuscript.

3. Figure legends, for both main figures and supplementary materials need major revisions; for example, repeated statements in figure S4. In addition, figure legends should follow the same format. Presented data should be adequately explained in figure legends, for example meaning of the arrowheads. All figures should be properly called in the text, for example, there is no reference for Figure 1F in the text, there are two figures S6, figure S3C, and entire figure S2 is incorrectly referenced.

Author response: We sincerely thank the referee for this scrutiny and apologise for the inconvenience caused by these errors. It was also pointed out by Referee #1. We attended each of the omissions listed above with appropriate corrections. We have now disengaged the combined legend of Fig 1F-G to two separate statements (Lines – 781-783) with a new description of figure 1F in the revised legend. We have also ensured that all figure panels are called at appropriate locations in the result section and described in the legend. The markings on each panel are defined in the legends, and statements are carefully revised to ensure that the data presented in each panel is described adequately in the legend. Also, the legends are reformatted to conform to a uniform pattern. We confirm that the revised legends only describe the panels and not the interpretations.

4. The authors should provide information regarding the number of experimental repeats used to perform statistical analysis and calculate significance.

Author response: We appreciate this comment. We have now indicated the N (number of antennae) and n (number of cilia/sensilla) values in the figures and legends as applicable.

5. All images, including zoomed panels should have scale bars.

Author response: We appreciate this comment. Missing scale bars are added in the concerned image panels with appropriate labels.

6. The authors have generated 5 mutant strains showing selective disruption of olfaction in adult flies, however, only 2 were used for subsequent studies. The authors should provide rationale for focusing on particular mutants.

Author response: We have made the necessary changes in the results section to justify using two out of the six alleles in the follow-up experiments as the following: “Since the jump response and EAG characteristics of these alleles appeared very similar, we decided to continue further phenotypic characterisations using the kj353 and kj925 alleles. Both these alleles have good homozygous viabilities.” (Line 194-196)

7. The authors use intensity heat maps to present data, the authors should specify whether they are uniform across images from different time points.
Author response: All images presented using the false-colour heat map across different time points or genetic backgrounds when placed within a figure panel were collected using identical laser power and gain setting to maintain comparability. This statement is added in the methods section (Line 451-453).

8. The authors state ‘We reasoned that Orco gene expression from 80-h APF [29] onwards could restrict the entry phase.’ This statement is unclear and should be rephrased.
Author response: We agree that the statement does not clearly express the intended conjecture. Therefore, we rephrased it as the following, “The onset of Orco expression, which begins from 80 hours APF [28], could determine the timing of its entry into the cilium.”

9. The authors find that one of the mutants with impaired olfaction used in this study have ‘[…] a small but significant reduction in the number of singlet microtubules […]’ yet the authors state that these results are consistent with lack of effect on tubulin level in the mutant cilia. Therefore, the interpretation of the data is unclear and should be adjusted.
Author response: We thank the reviewer for this feedback. We have revised the section to explain the observation with an appropriate interpretation as following: “The apparent difference in the microtubule count in the TEM sections and the total GFP:Tubulin84B levels assessed from the confocal images could be attributed to the difference in resolutions of these two techniques. Together, these results suggested that loss of kinesin-2 function in the adult olfactory cilia has a relatively stronger impact on Orco localisation than on the axoneme.” (Line 239-242)

We further showed that, “very little GFP:tubulin84B localised in the cilia when expressed using orcoGal4 as compared to the chaGal4 and or42bGal4, respectively (Figure S4B). OrcoGal4 expresses after 90 hours APF, much later than the onset of chaGal4 (30-34 hours APF) and or42bGal4 (60-70 hours APF). Hence, the results suggested that very little tubulin is transported into the cilia in the adult stage.” (Line 244-247)

10. The authors find that 2 used in this studies mutants with impaired olfaction show reduced localisation of Orco in cilia, however it is not clear what effect selected mutations have on Orco association with kinesin-2. Especially, that based on the presented data it appears that Orco’s level in cilia still increases over time, even in the mutant flies. Therefore, the authors should test whether the generated mutant show decrease in Orco-kinesin- interaction and/or impaired transport.

Author response: We truly appreciate this comment because we have debated on the issue ourselves and took some initiative to resolve the issue through experiments. We agree that the phenotype observed in the homozygous *Klp64D^kj353^* background could potentially be caused due to reduced affinity between the cargo and the motor. However, we believe that it is improbable because the same mutation eliminated KLP68D:YFP enrichment in the adult cilia (Figure 2E-F), indicating that it affects the motor stability/functions in the OSNs during the adult stage. We have provided extensive genetic characterisation data in Figures S3 and 3 to support the argument that the phenotype is specific to the olfactory system.

We understand the referee deduced from the data presented in Figure S4b in the first submitted version (Ref no. PGENETICS-D-21-00072), showing both the wild type and the Orco:GFP intensity increases with age. This experiment was performed using *orcoGal4>UAS-GFP:Orco* stock. However, a similar time-course analysis with the Endo-Orco:GFP did not elicit an age-dependent increment of the Orco:GFP fluorescence in the wild-type background (Figure S1F of the old and revised versions). Consistent with this argument, we now find that the Endo-Orco:GFP levels do not increase in the cilia beyond the 6 h stage in the homozygous *Klp64D^kj353^* background (Figures 4E and S5A the revised versions). Therefore, we concluded that the effect shown in the older version of Figure S4B could be an effect of the Gal4>UAS expression system. Gal4, being an exogenous protein, is relatively more persistent, which prolongs the target gene expression, and the presence of multiple UAS sequences enhances the expression of downstream genes. Although it raised another new interesting question regarding how increased and sustained Orco expression could enhance its localization in the cilia, the issue digressed form the focus and scope of the current manuscript. Therefore, we decided to remove the data from our consideration for this manuscript and investigate it in more detail later.

Unfortunately, while composing the supplemental data during the first submission, we made a mistake and forgot to remove the Figure S4A-B panels. We sincerely apologise for his sloppiness.

To answer whether the Orco transport is delayed in the *Klp64D^kj353^* homozygous and kinesin-2 RNAi backgrounds, we extended the time-course analysis of Endo-Orco:GFP enrichment in the cilia to 12 h AE (Figure 4E) and 6-days AE (Figure S5A) in the homozygous *Klp64D^kj353^* background, and up to 48 hours AE in the RNAi backgrounds (Figure 4E and S5B). We did not find any increment beyond 6 h AE in the first case, indicating that the Orco transport is not delayed in the *Klp64D^kj353^* background. The cilia morphology appeared degenerated from 24 h AE in the RNAi backgrounds (Figures S5B). Therefore, we could only compare EndoOrco:GFP levels at 12-h AE in the RNAi backgrounds (Figure 4E), which remained similar to corresponding 6 h AE values (Figure 4B, C). Together these observations suggested that the Orco transport is significantly reduced in the mutant background. Please note that the evidence does not suggest a delay.

**Therefore, the statement is revised by omitting the reference to ‘delayed transport’ from our discussion in the revised version of this manuscript.**

To further test whether the loss of kinesin-2 affects Orco movement inside the cilia, we conducted time-lapse imaging and a FRAP experiment at 5-minute intervals during the second episode of Orco transport at 4 h AE. We found that the loss of kinesin-2 significantly reduced the rate of recovery at the distal compartment of the cilia (Figure 4F and S6). We also found signatures of episodic increments of the Endo-Orco:GFP in the wild type cilia that lasted for less than 1 hour (Figure SB). The EndoOrco:GFP recovery is significantly reduced in the Kineins-2 RNAis and *Klp64D^kj353^* backgrounds (Figure 4F). This evidence suggested that kinesin-2 is also needed to manage Orco movement inside the cilia. Also, Orco diffusion along the ciliary membrane is restrained.

We could not enhance the time resolution of this experiment because of increased photobleaching at faster framerate. The olfactory cilia are housed within the cuticle which obstructed the signal form the cilia and contributed significant amount of background fluorescence. Hence, we had to use higher laser power than we would have liked.

In spite of these constraint, the additional results suggested that loss of kinesin-2 function affects the Orco transport into and inside the cilia.

Regarding measuring the affinity between Orco and Kinesin-2, we respectfully submit that it would be challenging to address this issue using the conventional pulldown assay. Primarily because the association should increase transiently during the transport episodes lasting for less than one hour each, and only a tiny fraction of the Orco present at the dendritic knob of the OSNs would participate in the process at a given time. Further, it is almost impossible to synchronise a fly culture to ensure a large number of eclosions without grossly altering the developmental programme. Therefore, one cannot obtain sufficient fly heads of the same age to perform this experiment. Besides, we have shown that the presence of Orco/ORx in a neuron does not facilitate its transport into the cilia, indicating that a third/fourth factor(s) would be involved. Hence, to begin with, one must identify these adaptors and determine how the affinity between them and kinesin-2 is controlled. It is beyond the scope of the current manuscript because a full-scale investigation with a new set of reagents would be necessary to effectively address the issue.

Regarding distinguishing a change in cargo-motor affinity from the disrupted transport, we respectfully beg to differ with the opinion expressed by the reviewer. According to our understanding, a reduction in the affinity would result in a loss of transport as much as that caused by a loss of motor activity. In both cases, one would find an accumulation of the cargo at the starting point and a reduction of the cargo at the destination. A defect in the motor function or weaker affinity with cargo will also distribute the cargo along the tracks. Hence, it would not be possible to discriminate between these two issues from the observable phenotypes. Most importantly, it would not result in a delayed transport. In our considered opinion, a delay in the transport can only happen if the signal that regulates the cargo-motor interaction is delayed. Such an effect is unlikely to be contributed by a mutation in the motor protein.

Finally, we would like to point out that the scope of this manuscript is to describe an independent role of heterotrimeric kinesin-2 in Orco transport into the cilia. We believe that the phenotypic characterisation at different time scales is well-correlated and consistent with this conclusion. In view of all these arguments and additional evidence presented in the revised version of the manuscript, we would respectfully request the referee to reconsider this suggestion.

11. Based on the presented data, it appears that signal of kinesin-2 only partially overlaps with that of Orco in cilia of olfactory neurons after eclosion (figure 6f). Given that presented measurements are based on the measurements of signal intensity over length of cilia in fly lines expressing GFP-tagged proteins of interest; it would be informative if the authors could supplement these observations with true colocalisation studies.

Author response: We appreciate this comment and thank the referee for the suggestion. We have added new colocalisation data in Figure 7G-H, which was obtained by expressing KLP68D:YFP in the Endo-Orco:GFP background. We used spectral imaging and liner-unmixing protocols to dissociate the GFP and YFP images. For this purpose, we used 445 nm to exclusively excite GFP and 514 nm to excite the YFP. The GFP emissions were obtained in the 495-515 band, and the YFP emission was collected in the 545-565 band. The emissions were collected in sequence using the lambda scanning protocol. Negligible YFP emission was detected in the 495-515 nm collection band when excited using 445nm. Similarly, the GFP emission was not detectable in the 545-565 nm band when excited using 514 nm. Please see the images below for reference.

Please note that the 445 nm excitation produces a strong emission from the cuticle in the 545-565 band, which was absent when excited using 514 nm. A similar result was obtained using the liner-unmixing protocol, but we chose to use the direct method for the colocalisation study to avoid compensation artefacts.

The functional kinesin-2 is a heterotrimeric complex and we had earlier shown that KLP68D can only move into the cilia in the presence of KLP64D (Jana et al., 2011) and KLP64D is unstable in the absence of KLP68D (Doodhi et al., 2012). Also, DmKAP (KAP3) was found to be essential for maintain the cilia (Sarpal et al., 2003; Jana et al., 2011), suggesting that KAP is an essential component of kinesin-2 in *Drosophila*. Therefore, we reasoned that KLP68D:YFP and KLP64D:GFP levels/distribution in the cilia would be limited by the physiological levels of the other subunits.

12. The authors show that distribution of Orco in kinesin-2 mutant and RNAi flies is not restricted to a ciliary sub-compartment, as in control flies after 6 hours following the eclosion. The authors however do not show whether in these backgrounds localisation of Orco to a ciliary domain is truly impaired or simply delayed.

Author response: We have now added a length-normalized plot profile of EndOrco:GFP at 12 hours AE (Figure S8-B-g). A careful examination of the profiles during 0-12 h AE in wild-type and mutant backgrounds revealed that Orco initially localizes in the designated domain and then ingresses towards the proximal end in the mutant backgrounds. The FWHM shift is clearly discernible at 6h and remained unchanged at 12 h AE. Therefore, it is clear that the transport is not delayed but truly impaired. Further, the FRAP data collected at a relatively higher time resolution supports the claim. Please note that the intensity profile is normalized along the length. Therefore, a delay in transport would have been reflected in movement of the FWHM of the plots towards the wild-type zone at a later time point. Exactly the opposite of what is observed.

13. The authors should include full western blots presented on figure 5 for evaluation.

Author response: The pulldown data presented in Figure 5 of the old version is redesignated as Figure 6 in the revised version. We have appended the original western blots used to compose Figures 6B, C and D below for inspection. We found that although a relatively higher level of GFP:Orco was pulled down with the GST-KLP68D-Tail fragment, a visible amount was also pulled down by the KLP64D-tail fragment. This evidence was suppressed in the main figure panel due to the transfer from one file format to another. We have now rectified the defect in the revised figure 6C. Accordingly, we also revised the statements related to Figure 6C in the results section to moderate the conclusion as the following: “a relatively more prominent level of GFP:Orco (Figure 6C) and OR47b, respectively, were pulled-down using the GST-KLP68D-Tail fragment (Figure 6D), which identified the potential Orco/ORx binding region at the tail domain of KLP68D (2β) motor subunit.”

Figure 6d: Loading order from the marker lane (right → left) : #1) input, #2) bead control, #3) GST, #4) GST-KLP68D-Tail, #5) GST-KLP64D-Tail
